# Identifying and Analyzing Novel Epilepsy-Related Genes Using Random Walk with Restart Algorithm

**DOI:** 10.1155/2017/6132436

**Published:** 2017-02-01

**Authors:** Wei Guo, Dong-Mei Shang, Jing-Hui Cao, Kaiyan Feng, Yi-Chun He, Yang Jiang, ShaoPeng Wang, Yu-Fei Gao

**Affiliations:** ^1^Department of Outpatient, China-Japan Union Hospital of Jilin University, Changchun 130033, China; ^2^Department of Neurosurgery, China-Japan Union Hospital of Jilin University, Changchun 130033, China; ^3^Department of Computer Science, Guangdong AIB Polytechnic, Guangzhou 510507, China; ^4^Department of Surgery, China-Japan Union Hospital of Jilin University, Changchun 130033, China; ^5^School of Life Sciences, Shanghai University, Shanghai 200444, China

## Abstract

As a pathological condition, epilepsy is caused by abnormal neuronal discharge in brain which will temporarily disrupt the cerebral functions. Epilepsy is a chronic disease which occurs in all ages and would seriously affect patients' personal lives. Thus, it is highly required to develop effective medicines or instruments to treat the disease. Identifying epilepsy-related genes is essential in order to understand and treat the disease because the corresponding proteins encoded by the epilepsy-related genes are candidates of the potential drug targets. In this study, a pioneering computational workflow was proposed to predict novel epilepsy-related genes using the random walk with restart (RWR) algorithm. As reported in the literature RWR algorithm often produces a number of false positive genes, and in this study a permutation test and functional association tests were implemented to filter the genes identified by RWR algorithm, which greatly reduce the number of suspected genes and result in only thirty-three novel epilepsy genes. Finally, these novel genes were analyzed based upon some recently published literatures. Our findings implicate that all novel genes were closely related to epilepsy. It is believed that the proposed workflow can also be applied to identify genes related to other diseases and deepen our understanding of the mechanisms of these diseases.

## 1. Introduction

As a classical neurological condition that may suddenly interrupt normal life activities and result in physical injury, epilepsy has been widely used to describe a group of epileptic seizure associated diseases [1, 2]. Epileptic seizures are the typical symptoms of the disease, which is the consequence of a disruption of the electrical communications between neurons [3]. As a common neurological disease, epilepsies are found all over the world and affect people of all ages [4]. Only in America, more than one hundred thousand incident cases are diagnosed as epilepsy per year, seriously threatening their mental and physical health [5, 6]. Nowadays, the clinical study on epilepsy has been progressively deepening and typical diagnosis routines of epilepsy have been set up. Considering that epileptic seizures induced by disruption of electrical communications between neurons are the typical symptoms of epilepsy, the long-term paroxysmal epileptic seizures might suggest the initiation and progression of such neurological disease [7, 8]. Generally, it is quite necessary to turn to the doctor for help after someone has more than twice abnormal seizures excluding those with known medical conditions. Considering epilepsy has many subtypes induced by different pathogenic factors, resulting in different complications with similar early seizures symptoms, the diagnosis of epilepsy contributes not only to a confirmation of epilepsy but also to classification of the epilepsy from which the patients suffer into its subtype [9, 10]. The diagnosis of epilepsy can be divided into two main procedures: medical history taking and instrumental inspections. Previous experiments have confirmed that typical family history and multiple medical conditions may lead to neurological abnormalities, which may contribute to the initiation and progression of epilepsy [11–13]. Therefore, the first step of the diagnosis of epilepsy is to inquire about the medical history of the patients and their respective family. However, a definitive diagnosis of epilepsy is performed by the following instrumental inspections. As we have mentioned above, epilepsy is referred to as a group of neurological diseases induced by abnormal electrical communication between neurons [4]. Therefore, the measurement of electrical impulses in brain by an electroencephalogram (EEG) test has been regarded as one of the golden standards for epilepsy diagnosis [14, 15]. Apart from EGG, magnetic resonance spectroscopy (MRS), positron emission tomography (PET), and magnetic resonance imaging (MRI) have also been widely used to diagnose epilepsy [16–18].

Although great progresses have been made to diagnose epilepsy, the therapeutic methods to treat epilepsy are still quite limited and they mainly contribute to the symptomatic relief. There are two main functional methods to relieve the seizure symptoms of epilepsy: certain nutrient intakes and vagus nerve stimulations by surgery [19–22]. The therapeutic nutrients that have been confirmed to contribute to the relief of epilepsy include folic acid, melatonin, and vitamins (large doses) [19]. However, such a treatment cannot provide a permanent cure but tries to temporarily relieve the symptoms. The vagus nerve stimulation, as the most effective treatment for epilepsy, necessitates implantation of a pacemaker-like device in the patient's body to stimulate the vagus nerve, relieving the symptoms with few side-effects [23]. These treatments do not have an in-depth consideration of the pathogenic factors that cause the disease but mainly concentrate on the relief of the seizure symptoms. To develop more effective curing methods, it is quite fundamental to understand and reveal the pathogenesis of epilepsy.

As it is known, traditionally epilepsy is referred to a group of diseases characterized by similar symptoms (i.e., epileptic seizures), but not by its pathogenesis. And the underlying pathogenesis of epilepsy may be quite complicated. In the past, due to technological constraints, the pathogenesis of epilepsy is largely unknown. It has been reported that the occurrence of some epilepsy cases turned out to exhibit some degree of familial aggregation, not only implicating the significance of history taking, but also suggesting that the genetic background contributes to the disease [24, 25]. According to some clinical data, it is without any doubt that if a sibling suffers from epilepsy, the brothers and sisters who have similar genetic background inherited from their patients are at higher risk of epilepsy comparing to those who do not [26, 27]. However, the detailed pathogenesis cannot be clearly revealed. Recently, with the development of sequencing technologies, some epilepsy associated genes with either pathological mutations or copy number variants have been identified [28, 29]. Among these genes, a group of functional genes, the sodium channel protein family, encode the core sodium channel in the nerve system [30]; for example, genes like SCN1A and SCN8A encoding core component of the sodium channel have been confirmed to be associated with the progression of hereditary epilepsies [31–33]. Therefore, specific genes may play a definitive role during the initiation and progression of the epilepsy, as hereditary epilepsies are deemed to have a certain genetic background.

Although specific genes have been strongly suggested to be associated with epilepsy, however, it is quite hard to identify the core regulatory genes related to epilepsy by time-consuming experimental methods such as the western blot [34, 35]. Here, based on some known epilepsy-related genes, we presented a new computational workflow to search out potential genes of interest. The random walk with restart (RWR) algorithm was employed in our workflow to search possible novel genes in a protein-protein interaction (PPI) network. Compared to the network methods based on guilt-by-association [36] which only consider the neighbors of known genes [37–39], the RWR algorithm can inspect the whole network to make extensive decisions; that is, methods based on guilt-by-association used part of the network, while the RWR algorithm can utilize the whole network. The brief procedures of our workflow was described as follows. Firstly, the RWR algorithm was executed on a PPI network using validated epilepsy associated genes as seed nodes and genes receiving high probabilities were selected as possible candidate genes. Then, these possible genes were screened by a permutation test, followed by functional association tests, resulting in thirty-three novel epilepsy-related genes. Further analysis indicates that all genes obtained may directly or indirectly contribute to the initiation and progression of epilepsy. To the best of our knowledge, this is the first study attempting to identify core regulatory factors of epilepsy using computational methods. These newly found genes may reveal the underlying mechanisms of epilepsy, and the approach may be extended to solve the similar problems of other complex diseases.

## 2. Materials and Methods

### 2.1. Epilepsy Related Genes

499 genes related to epilepsy were retrieved from EpilepsyGene (http://61.152.91.49/EpilepsyGene/download.php) [40], a genetic resource for genes and their mutations related to epilepsy. The epilepsy genes in EpilepsyGene database were collected by searching the PubMed database (https://www.ncbi.nlm.nih.gov/pubmed). Because our method was executed on a PPI network (referred to in Section 2.2), all 499 genes were linked to their Ensembl IDs. Those without Ensembl IDs or those Ensembl IDs do not occur in the PPI network were excluded. Finally, 470 genes with their Ensembl IDs were obtained for investigation in this study. The detailed information of these genes is provided in Supplementary Material S1 in Supplementary Material available online at https://doi.org/10.1155/2017/6132436.

### 2.2. PPI Network

The interactions between proteins within and outside the cells provide useful information about their activities, properties, and functions. Two proteins that can interact with each other produce a PPI (protein-protein interaction), which often share similar functions or involve in the same biological processes. The PPI network comprised of large amounts of PPIs representing proteins' complicated interaction relationships and remote functional relationships in signaling pathways, such as the proteins involved in regulation and catalysis activity in glycolysis and tricarboxylic acid cycle [41–43]. Some computational predictors and workflows employed PPIs to predict protein functions [44–46] and search for novel genes related to a variety of diseases [47–51]. Therefore, PPIs could be useful to infer novel epilepsy-related genes based on the validated 470 epilepsy genes mentioned in Section 2.1.

To obtain the PPI information and construct a PPI network, the human PPI information was retrieved from STRING (Version 10.0, http://string-db.org/) [52], a well-known public database collecting known and predicted protein-protein interactions. In the current version, it covers 9,643,763 proteins from 2,031 organisms. Interactions reported in STRING are derived from the following five sources: (I) Genomic Context Predictions; (II) High-throughput Lab Experiments; (III) (Conserved) Co-Expression; (IV) Automated Text mining; (V) Previous Knowledge in Databases. The human PPIs are collected in the file “9606.protein.links.v10.txt.gz” that can be accessed from the download page of STRING using “Homo sapiens” as a restriction to the data. Accordingly, we obtained 4,274,001 human PPIs covering 19,247 proteins. Because the 4,274,001 human PPIs include not only direct (physical) but also indirect (functional) interactions between proteins, these PPIs can offer relatively more information about the novel genes related to epilepsy.

For each PPI, there are two Ensembl IDs representing two proteins and a score ranging from 150 to 999 that indicates the strength of the interaction. A larger score assigned to a PPI indicates that the two proteins are more likely to interact with each other. For proteins *p*_*a*_ and *p*_*b*_, their interaction score was denoted as *S*(*p*_*a*_, *p*_*b*_). In the network, the 19,247 proteins were denoted as the nodes and two proteins were connected by an edge if and only if they can form a PPI. Thus, there were 4,274,001 edges in the network, and each edge represented a PPI. In addition, the interaction score was added to the network as the weight of the corresponding edge. For convenience, the PPI network was denoted as *G* in the following sections.

### 2.3. RWR Algorithm

As a ranking algorithm, the RWR algorithm simulated a walker starting from a seed node or several seed nodes and randomly moved on the network* G *[53]. In this study, 470 Ensembl IDs of epilepsy genes were set as the seed nodes. Based on them, we aim to mine some potential genes functionally related to epilepsy. In the beginning of the algorithm, an initialization vector *P*_0_ was constructed with 19,247 components in it and each component was a score rating the probability of each node being a potential epilepsy-related gene. The probability scores of 470 Ensembl IDs that represented validated epilepsy genes in *P*_0_ were set to 1/470 (0.0021) and other components were set to zeros. If the vector *P*_*i*_ was the probability vector after the RWR algorithm was executed *i*th round, then the iteration equation can be formulated as follows:

(1) $$\begin{array}{c} P_{i+1}=\left(\;1-r\;\right)A^{T}P_{i}+rP_{0}, \end{array}$$

where *A* was the column-wise normalized adjacency matrix and *r* was the probability that it returned to the start nodes, which was set to 0.8 in this study. When probability vector *P*_*i*+1_ and *P*_*i*_ satisfy the inequality ‖*P*_*i*+1_ − *P*_*i*_‖_*L*_1__ < 1*E* − 06, the iteration stopped and *P*_*i*+1_ was output as the results of the RWR algorithm.

According to the probability vector yielded by RWR algorithm, each node (gene) in the network was given a number representing the probability of it being a novel epilepsy gene. Genes with larger values are more likely to be epilepsy-related genes. Threshold 1*E* − 05 was adopted in this study; that is, genes receiving scores larger than 1*E* − 05 were selected from the network* G*, because it filtered out a large portion of genes and there remained enough genes for further analysis. For convenience, the obtained genes were called RWR genes.

### 2.4. Filtering Methods

After RWR algorithm was executed on the network, many RWR genes could be selected. However, there are likely many false positive genes among them as elaborated in our previous study [44]. These genes are not special to the epilepsy and should be excluded. In this section, a two-step filtering method was proposed to screen out false positive genes.

#### 2.4.1. Permutation Test

The structure of network *G* can influence the output of RWR algorithm, which may lead to the false selection of some RWR genes. For example, a node with a degree higher than average degree of the network *G* may receive a larger score by RWR algorithm even if it was not related to epilepsy. To mine this type of nodes in the network, a permutation test was applied on the network. Firstly, 1,000 Ensembl ID sets, denoted as *S*_1_, *S*_2_,…, *S*_1000_, were randomly produced and each set contained 470 random Ensembl IDs. Secondly, for each set, the RWR algorithm was executed on the network *G* using the 470 Ensembl IDs in this set as seed nodes, thereby yielding a probability for each RWR gene. Thirdly, for each RWR gene *g*, a measurement, namely, permutation FDR, was calculated based on the following equation:

(2) $$\begin{array}{c} FDR\left(\;g\;\right)=\frac{\Theta }{1000}, \end{array}$$

where Θ was the number of randomly produced sets in which the score of gene *g* was larger than the score computed by the validated epilepsy related genes. According to (2), the higher permutation FDR an RWR gene had, the less possible the gene was an epilepsy related gene. Because 0.05 was widely used as a common cutoff in statistical test, it was also set to be the threshold of permutation FDR in this study. Therefore, the RWR genes with permutation FDRs less than 0.05 were selected and called candidate genes for further analysis.

#### 2.4.2. Functional Association Test

Among the candidate genes, some of them were functionally highly associated with epilepsy while others weakly associated with it. To select essential genes among them, a functional association test that consisted of two selection schemes was proposed.

It is known that proteins in a PPI with a higher interaction score are more likely to share similar functions. Among the candidate genes, those having strong associations with validated epilepsy-related genes could be the most likely novel epilepsy genes. If a candidate gene has strong associations with exact one validated epilepsy-related gene and has weak or no associations with other epilepsy-related genes, it may still be a novel epilepsy-related gene. In view of this, we believe that using the associations between a candidate gene and its most related epilepsy-related gene is more proper to indicate its associations with epilepsy. Accordingly, an interaction measurement called maximum interaction score (MIS) was calculated for each candidate gene *g*, which can be defined as

(3) MISg=max⁡Sg,g′:  g′  is  an  epilepsy-related  gene.

Candidate genes with large MISs mean that it is highly possible that they can directly interact with at least one validated epilepsy gene and may cause the symptoms of epilepsy. In STRING, the value 900 is set to be the cutoff to achieve a highest confidence. Thus, it was also set to be the threshold of MIS; that is, candidate genes with MISs less than 900 were discarded.

Besides, genes related to epilepsy may share some common gene ontology (GO) [54] terms and often occurred in the same Kyoto Encyclopedia of Genes and Genomes (KEGG) [55] pathways. Thus, candidate genes sharing same or similar GO terms and KEGG pathways with validated epilepsy genes are more likely to be the genes related to epilepsy. The enrichment theory of GO terms (KEGG pathways) [56–58] was used to quantitatively measure the relationship between a gene and GO terms (KEGG pathways). For a gene *g*, let us denote the set containing *g* and its direct neighbor genes in the PPI network reported in STRING by *H*(*g*). Then, the relationship between *g* and one GO term or KEGG pathway can be encoded into a numeric value as follows:

(4) $$\begin{array}{c} S_{GO}\left(\;g,G\;\right)=-log_{10}\left(\;\overset{n}{\underset{k=m}{\sum }}\frac{\left(\begin{array}{c} M\\ k \end{array}\right)\left(\begin{array}{c} N-M\\ n-k \end{array}\right)}{\left(\begin{array}{c} N\\ n \end{array}\right)}\;\right), \end{array}$$

where *G* represented a GO term or a KEGG pathway, *N* was the total number of genes in humans, *M* was the number of genes annotated to *G*, *n* was the number of genes in *H*(*g*), and *m* was the number of genes that are in *H*(*g*) and annotated by *G*. The values for all GO terms and KEGG pathways can be collected into a vector *ES*(*g*). The similarity of two genes *g* and *g*′ on GO terms and KEGG pathways can be measured by the proximity of the two vectors *ES*(*g*) and *ES*(*g*′) as follows:

(5) $$\begin{array}{c} \Gamma \left(\;g,g^{\prime }\;\right)=\frac{ES\left(g\right)\cdot ES\left(g^{\prime }\right)}{\left\|ES\left(g\right)\right\|\cdot \left\|ES\left(g^{\prime }\right)\right\|}. \end{array}$$

It is clear that if the resultant number of (5) is large, *g* and *g*′ are similar on GO terms and KEGG pathways, implicating a strong relationship between them. With similar arguments on MIS, for each candidate gene *g*, the maximum function score (MFS) was calculated as follows:

(6) MFSg=max⁡Γg,g′:  g′  is  an  epilepsy-related  gene.

A candidate gene receiving a high MFS means it shares relatively more GO terms and KEGG pathways with at least one validated epilepsy gene. In this study, we tried 0.9 as the threshold of MFS; that is, candidate genes with MFSs larger than 0.9 were selected.

In short, candidate genes resulting from permutation test with MISs larger than or equal to 900 and MFSs larger than 0.9 were selected. For convenience, they were named as core candidate genes.

## 3. Results

**Figure 1:**
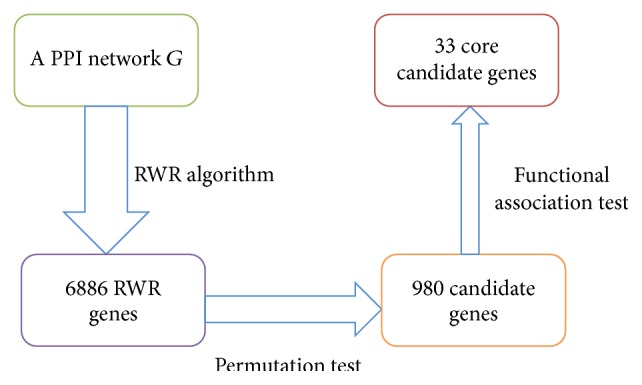
The flowchart of RWR algorithm and filtering methods for identifying core candidate genes.

An outline for the procedure of the method, including RWR algorithm and filtering methods described in Sections 2.3 and 2.4, by a flowchart is illustrated in Figure 1. This section would show the detailed results yielded by the method.

As described in Section 2.3, the RWR algorithm was executed on the PPI network* G*, in which the 470 Ensembl IDs were used as seed nodes. A probability vector can be obtained, in which each composition represents the probability score of the corresponding node (gene) being a novel epilepsy-related gene. Genes with probabilities larger than 1*E* − 05 were selected, producing 6,886 RWR genes, which are listed in Supplementary Material S2.

For the 6,886 RWR genes derived from RWR algorithm, a permutation test was applied on them to screen out RWR genes that are not special for epilepsy. The permutation FDR was calculated for each RWR gene, which is provided in Supplementary Material S2. Value 0.05 was set to be the threshold of permutation FDR, thereby producing 980 candidate genes, which are listed in Supplementary Material S3.

To further select genes that are functionally related to epilepsy, a functional association test was applied to the 980 candidate genes. As described in Section 2.4.2, for each candidate gene, we calculated its MIS (cf. (3)) and MFS (cf. (6)). Values 900 and 0.9 were used as the threshold of MIS and MFS, respectively. And finally thirty-three core candidate genes were obtained. These genes were deemed to be closely related to epilepsy and are listed in Table 1. According to some recent published literature as discussed in Section 4, these core candidate genes, which had similar functions with the validated genes, are highly likely to be the novel epilepsy genes.

## 4. Discussions

**Figure 2:**
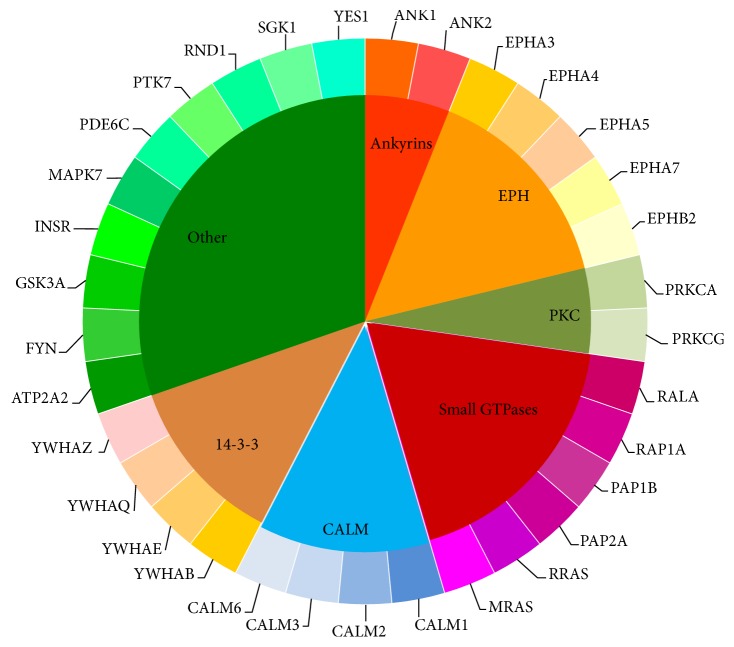
The distribution of the thirty-three core candidate genes according to their protein families.

For a long time, epilepsy has been regarded as complicated neurological diseases with various pathogenesis. Based on clinical data, the occurrence of epilepsy has shown conspicuous familial aggregation characteristics, implying that genetic background features (such as mutations, copy number variants of genes) may play an irreplaceable role for epilepsy [59–61]. Recent publications have also confirmed such implication. Various epilepsy associated genes have been identified [62–66]. However, it is quite expensive and time-consuming to identify epilepsy associated genes with experiments. Based on our computational method, we identified thirty-three candidate epilepsy associated genes, listed in Table 1. According to some recent publications, all these core candidates show specific relationship with the initiation and progression of epilepsy, validating the effectiveness of our computational method. According to the gene families of these thirty-three core candidate genes, we classified them into seven clusters, shown in Figure 2, and analyzed them accordingly.

*Ankyrins*. In our prediction list, two of the candidate genes,* ANK1* and* ANK2,* turned out to be the functional members of the ankyrins. As we all know, ankyrins are a group of connexin that link the integral membrane proteins to the cytoskeleton, which have been widely reported that they contribute to cell proliferation, motility, and the maintenance of specialized membrane domains [67–69]. In human bodies, ankyrins have been confirmed to bind to the voltage-gated potassium channel subunits KCNQ2 and KCNQ3, regulating their normal functions [70]. Considering that KCNQ2 and KCNQ3 turned out to directly contributing to the initiation and progression of epilepsy, our predicted genes ANK1 and ANK2 as the functional components of ankyrins may very probably be epilepsy associated genes, validating our prediction [71]. 

*EPH Subfamily*. Apart from the Ankyrin protein family, another group of proteins, the EPH subfamily, have also been identified to contribute to epilepsy. In our prediction list, five genes can be classified in such subfamily:* EPHA3*,* EPHA4*,* EPHA5*,* EPHA7,* and* EPHB2*. Such five genes all encode the receptors for the erythropoietin-producing hepatoma amplified sequences (EPH), acting as the tyrosine-protein kinase receptor [72, 73]. In mouse model, it has been confirmed that the activation of EPH receptor associated genes, like EPHB3, contributes to the onset of epilepsy [74]. Considering the functional similarity and underlying correlations, it is quite reasonable that our predicted genes of EPH receptor family may also contribute to such processes [75]. Apart from that, another publication confirmed that, by stimulating NMDA receptor activity, ERK activates the progression of epilepsy [76]. During the activation, various genes of our predicted EPH family have been identified to promote such biological processes, validating the crucial role of EPH family including our predicted genes EPHA3, EPHA4, EPHA5, EPHA7, and EPHB2 during epilepsy.

*Protein Kinases*. Two of our predicted candidates,* PRKCA* and* PRKCG,* can be clustered into another functional family, the family of serine- and threonine-specific protein kinases. Such two genes turn out to be functional components of the protein kinase C, a core member of the protein family we mentioned above [77–79]. The protein kinase C associated signaling pathway has been widely reported to be associated with epilepsy and may be a candidate therapeutic target for such disease [80]. As two major components for such pathway, our predicted genes PRKCA and PRKCG may definitely contribute to such disease. Apart from such evidence, a specific mutation of PRKCG (SCA-14) has been reported to be associated with a typical movement disorder, which can be called Ramsay Hunt phenotype [81]. Considering that such disorder has been widely identified in epilepsy patients, such mutation may be functionally related to the progression of epilepsy, validating our prediction [82, 83]. As for MAPK7, such gene has been widely regarded as a multifunctional gene that involves various biological processes including proliferation, differentiation, transcription regulation, and development [84]. MAPK7 has been reported to interact with a specific protein Aquaporin 4 (AQP4) in human beings [85]. Since AQP4 has been confirmed to accumulate in neuron cell during epilepsy and contribute to the pathological processes, it is quite reasonable to summarize that, as a functional related protein of AQP4, our predicted gene MAPK7 very probably contributes to epilepsy, validating the accuracy and efficacy of our prediction [85]. The next gene is also a crucial kinase for human beings, the* PTK7*. Although, different from other proteins from protein tyrosine kinase family, PTK7 lacks detectable catalytic tyrosine kinase activity, it has still been reported to contribute to the functional Wnt signaling pathway and regulate the cellular polarity and adhesion [86]. Though no direct relationship between PTK7 and epilepsy has been confirmed, recent publications reported that PTK7 may participate in the metabolism of antiepileptic drugs (AED), suggesting that there may remain uncovered interactions between PTK7 and epilepsy, validating our prediction [87].* GSK3A*, as a multifunctional Ser/Thr protein kinase, has been reported to contribute to glycogen synthesis and transcriptional regulation [88, 89]. Such gene has been reported to be quite essential for the development and maturation of cortical neurons [90]. Considering that cortical neurons, especially the migration of neurons, are quite significant for epilepsy, our predicted gene GSK3A may very probably be epilepsy associated gene [91, 92].

*Small GTPases*. Apart from PKC associated genes, there are also six genes (*RALA*,* RAP1A*,* RAP1B*,* RAP2A*,* RRAS,* and* MRAS*) that can be clustered into the famous Ras family of small GTPases. Based on recent publications, various small GTPases have been identified to contribute to the progression of epilepsy, including Cdc42, RAB39B [93, 94]. As for our predicted candidates, it has been confirmed that, during the pathological processes of epilepsy, the normal function of RAP1A and its related Ras signaling pathway has been regulated and altered by microRNAs, implying the potential role of Ras signaling pathway during epilepsy [95]. As for RAP1B and RAP2A, RAP1B has been confirmed to be specifically activated in nerve system and contributes to the regeneration of neuronal connectivity, the dysfunction of which turns out to be one of the pathological factors for epilepsy, validating the regulatory role of RAP1B during such disease [96]. RAP2A has been validated and confirmed to contribute to the childhood absence epilepsy, a specific subtype of epilepsy, and may be related with a specific glioma inducing epilepsy associated symptoms, validating our prediction of epilepsy associated genes [97, 98]. As for RRAS and MRAS, considering the functional similarity of MRAS and MAPK, the detailed analysis of such genes can be seen below, while the inner relationship between RRAS and epilepsy has also been revealed in mouse model, validating our prediction [99, 100]. RALA, encoding a functional small GTPase belonging to Ras family, has been confirmed to mediate the transmembrane signaling by the occupancy of functional receptors [101]. Such gene has been definitely confirmed to be associated with epilepsy by regulating the drug resistance of such disease [102]. Another gene,* RND1,* encodes a small GTPase, which does not belong to Ras family but to Rho GTPase family. In response to various extracellular signaling, the protein encoded by such gene turns out to regulate the actin cytoskeleton [103, 104]. In intractable epilepsy, a clinical subtype of epilepsy which is hard to cure, recent publications confirmed the expression of such gene in the central nerve system of the patients, implying that such gene may definitely contribute to the progression and prognosis of such disease [105, 106]. MAPK7 and MRAS are two proliferation-associated genes in our candidate epilepsy associated gene list. Among them,* MRAS* turn out to contribute to Ras signaling pathway, which has been confirmed above to be associated with the progression of epilepsy [95]. What is more, as for MRAS itself, it has been reported that such gene may contribute to the development of brain in early stage and the abnormal activation of such gene may induce epilepsy-like syndrome, validating our prediction [107].

*Calmodulin (CALM) Family*. Apart from that, such genes may also contribute to the specific seizure like features during the progression of epilepsy, suggesting its core regulatory role [108]. Four genes (*CALM1*,* CALM2*,* CALM3,* and* CALM6*) of the functional calmodulin (CALM) family have also been predicted to contribute to epilepsy. Genes of calmodulin family mainly act as a calcium binding protein that participate in cell cycle and proliferation associated biological processes [109, 110]. Considering that epilepsy has been confirmed to be associated with abnormal calcium ion transportation, it is quite reasonable to speculate that our predicted genes of CALM family may contribute to epilepsy which has also been confirmed by recent publications [111, 112]. 

*14-3-3 Family of Proteins*. The remaining group of functional proteins, including* YWHAB*,* YWHAE*,* YWHAQ*, and* YWHAZ*, that contribute to epilepsy turn out to be encoded by the so-called 14-3-3 family of proteins. Such family of proteins contribute to the signaling transduction by binding to phosphoserine-containing proteins [113]. As we have mentioned above, epilepsy has been confirmed to be associated with the protein kinase C signaling pathway [81]. Recent publications identified that, during the progression of epilepsy, our predicted candidates, proteins of 14-3-3 family, may interact with protein kinase C and further promote the progression, implying the functional role of such genes [114]. Apart from that, such five genes that we sorted out have also been directly identified in epilepsy cases. Take YWHAB as an example. Such gene has been identified to contribute to the regeneration of neurons after physical or chemical injury. The dysfunction of such gene may be related to the initiation of epilepsy in certain pathological conditions [115]. Therefore, such four genes in our prediction list have all been confirmed to participate in epilepsy associated biological processes, validating the accuracy and efficacy of our prediction.

*Other Crucial Genes*. Apart from such genes, there still remain six genes with no clear family enrichment in our prediction list that may also contribute to epilepsy in their respective ways. Among such genes,* ATP2A2* turns out to encode a significant intracellular pump located in the sarcoplasmic or endoplasmic reticula [116, 117]. Epilepsy has been confirmed to be a specific complication of various diseases, including Darier's disease [118, 119]. Our predicted gene ATP2A2 has been identified in clinical cases of Darier's disease and may directly contribute to epilepsy associated symptoms, validating the efficacy and accuracy of our prediction [120]. Another gene* INSR* turns out to be the functional receptor for a core endogenous hormone insulin, which further activates the downstream of insulin signaling pathway [121, 122]. As for the underlying relationship between INSR and epilepsy, it has been reported that a group of specific mutations in INSR: INSR H1085H C>T, G972R has been confirmed to specifically occur in the epilepsy patients in Han Chinese, validating the specific role of INSR during the progression of epilepsy [123]. Another gene* FYN* is also a candidate oncogene just like genes from Ras family as we have mentioned above [124, 125]. Recent publications reveal the potential relationship between our predicted gene FYN and amygdala kindling, a specific phenotype associated with epileptogenesis [126]. Participating in mTOR signaling pathway, though the detailed mechanism of the pathology of FYN initiated epilepsy has not been fully revealed, our predicted gene FYN may definitely be an epilepsy associated gene [126–128].

Phosphodiesterase 6C, encoded by our predicted gene* PDE6C,* has been confirmed to contribute to pyrimidine metabolism and phototransduction [129, 130]. Autosomal-dominant cerebellar ataxia is a specific symptom of epilepsy in human beings [131]. It has been reported that a specific mutation, p.Arg95His, of our predicted gene, PDE6C, may be associated with the autosomal-dominant cerebellar [132]. Considering the inner linkage between autosomal-dominant cerebellar ataxia and epilepsy in human beings, our predicted gene PDE6C may also contribute to the progression of epilepsy, validating our prediction [131]. As we have mentioned above, quite a lot of kinases have been reported to contribute to the abnormal biological process during epilepsy. The remaining two genes* SGK1* and* YES1* have also been suggested to contribute to the progression of epilepsy. SGK1 turns out to encode a serum/glucocorticoid regulated kinase, which further contributes to the regulation of cellular stress response [133, 134]. It has been confirmed that our predicted gene SGK1 is upregulated by a functional cellular component, aldosterone [134]. It has been confirmed that aldosterone has strong chemical and biological effects on epileptic seizures, implying that our predicted gene SGK1 may contribute to the regulation of the typical symptoms of epilepsy, the seizures [135]. Therefore, SGK1 very probably is a functional epilepsy associated gene. The last gene, YES1, also encodes a small GTPase that has been widely regarded as a tumor associated gene [136]. Although no direct report confirms the relationship between YES1 and epilepsy, considering the core regulatory role of small GTPases for epilepsy and that it has been confirmed that YES1 is expressed in brain and central nerve system, it is quite reasonable for us to believe that YES may be a functional epilepsy associated gene [137].

## 5. Conclusion

Based on our newly developed computational method, we have identified thirty-three novel genes that may contribute to the initiation and progression of epilepsy. According to the comprehensive analyses on these genes, they are strongly suspected to be either directly or indirectly related to epilepsy, validating the effectiveness of our method. In summary, this method can not only contribute to the identification of potential epilepsy associated genes but also provide a new tool to investigate the underlying mechanisms of the pathological processes of epilepsy.

## Supplementary Material

The Supplementary Material consists of three files. In detail, Supplementary Material S1 lists 470 genes related to epilepsy with their Ensembl IDs and gene symbols; Supplementary Material S2 lists 6,886 RWR genes derived from RWR algorithm with probability larger than 1E-05; Supplementary Material S3 lists 980 candidate genes with permutation FDRs less than 0.05.

## Figures and Tables

**Table 1:** Thirty-three core candidate genes identified by our method.

Gene symbol	Ensembl ID	Probability	Permutation FDR	MIS	MFS
ANK2	ENSP00000349588	4.53*E* − 05	<0.001	990	0.997
ANK1	ENSP00000265709	3.52*E* − 05	0.034	995	0.995
EPHA7	ENSP00000358309	3.28*E* − 05	0.025	906	0.988
EPHA5	ENSP00000273854	3.46*E* − 05	0.032	906	0.988
PRIKCG	ENSP00000263431	4.75*E* − 05	0.025	905	0.987
PTK7	ENSP00000230419	3.10*E* − 05	0.023	943	0.987
EPHA3	ENSP00000337451	4.40*E* − 05	0.017	912	0.986
PDE6C	ENSP00000360502	3.61*E* − 05	0.032	900	0.980
EPHA4	ENSP00000281821	4.16*E* − 05	0.008	990	0.980
YWHAQ	ENSP00000238081	5.50*E* − 05	0.004	999	0.978
GSK3A	ENSP00000222330	6.40*E* − 05	0.017	977	0.976
CALM1	ENSP00000349467	6.55*E* − 05	0.023	966	0.976
EPHB2	ENSP00000363763	4.39*E* − 05	0.026	908	0.975
PRIKCA	ENSP00000408695	7.02*E* − 05	0.039	992	0.975
CALM2	ENSP00000272298	5.52*E* − 05	0.048	985	0.974
YWHAE	ENSP00000264335	1.63*E* − 04	0.009	999	0.973
YWHAB	ENSP00000300161	5.56*E* − 05	0.047	999	0.971
ATP2A2	ENSP00000440045	5.03*E* − 05	0.005	908	0.970
YES1	ENSP00000324740	6.54*E* − 05	0.002	967	0.969
CALML3	ENSP00000315299	3.96*E* − 05	0.027	906	0.968
SGK1	ENSP00000356832	4.90*E* − 05	0.034	999	0.966
CALML6	ENSP00000304643	4.44*E* − 05	0.01	909	0.965
YWHAZ	ENSP00000309503	1.86*E* − 04	0.002	999	0.958
MAPK7	ENSP00000311005	7.15*E* − 05	0.031	999	0.956
RRAS	ENSP00000246792	5.94*E* − 05	0.004	951	0.941
RAP2A	ENSP00000245304	4.97*E* − 05	0.021	940	0.937
RALA	ENSP00000005257	6.57*E* − 05	0.006	981	0.932
RAP1B	ENSP00000250559	5.28*E* − 05	0.009	972	0.931
MRAS	ENSP00000289104	4.70*E* − 05	0.022	932	0.931
INSR	ENSP00000303830	7.27*E* − 05	0.024	996	0.927
PAP1A	ENSP00000348786	6.49*E* − 05	0.014	995	0.925
RND1	ENSP00000308461	5.70*E* − 05	0.002	996	0.903
FYN	ENSP00000346671	1.02*E* − 04	<0.001	999	0.900
